# Natural Fibers as an Alternative to Synthetic Fibers in Reinforcement of Geopolymer Matrices: A Comparative Review

**DOI:** 10.3390/polym13152493

**Published:** 2021-07-28

**Authors:** Afonso R. G. de Azevedo, Ariana S. A. Cruz, Markssuel T. Marvila, Leandro B. de Oliveira, Sergio N. Monteiro, Carlos Mauricio F. Vieira, Roman Fediuk, Roman Timokhin, Nikolai Vatin, Marina Daironas

**Affiliations:** 1 LECIV—Civil Engineering Laboratory, UENF—State University of the Northern Rio de Janeiro, Av. Alberto Lamego, 2000, Campos dos Goytacazes 28013-602, RJ, Brazil; ariana.sazeredo@gmail.com; 2 LAMAV—Advanced Materials Laboratory, UENF—State University of the Northern Rio de Janeiro, Av. Alberto Lamego, 2000, Campos dos Goytacazes 28013-602, RJ, Brazil; markssuel@hotmail.com (M.T.M.); leandrobarbosa850@gmail.com (L.B.d.O.); vieira@uenf.br (C.M.F.V.); 3 Department of Materials Science, IME—Military Institute of Engineering, Square General Tibúrcio, 80, Rio de Janeiro 22290-270, RJ, Brazil; snevesmonteiro@gmail.com; 4 Polytechnic Institute, Far Eastern Federal University, 690922 Vladivostok, Russia; timokhin.ra@students.dvfu.ru; 5 Peter the Great St. Petersburg Polytechnic University, 195251 St. Petersburg, Russia; vatin@mail.ru; 6 North Caucasus Branch, Belgorod State Technological University Named after V.G. Shukhov, 357202 Mineralnye Vody, Russia; cafedrapzgsh@yandex.ru

**Keywords:** natural fibers, synthetic fibers, geopolymer matrices

## Abstract

Geopolymer materials have been gaining ground in the civil construction sector not only for having superior physical properties when compared to conventional cement, but also for being less harmful to the environment, since the synthesis of the geopolymer does not release toxic gases or require high energy costs. On the other hand, geopolymer materials like cementitious matrices have low flexural strength and have fragile breakage. To overcome these deficiencies, the insertion of fibers in geopolymeric matrices has been evaluated as a solution. Although most research on this practice focuses on the use of synthetic fibers, the use of natural fibers has been growing and brings as an advantage the possibility of producing an even more ecological material, satisfying the need to create eco-friendly materials that exists today in society. Thus, this paper aimed to, through the evaluation of research available in the literature, understand the behavior of fibers in geopolymer matrices, identify similarities and differences between the performance of geopolymer composites reinforced with natural and synthetic fibers and, understanding that it is possible, point out ways to optimize the performance of these composites.

## 1. Introduction

Geopolymers are inorganic polymers formed by the polymerization of aluminosilicate precursors (such as fly ash, metakaolin, and blast furnace slag) in an alkaline solution [1]. Geopolymer composites are today seen as excellent alternatives to Portland cement-based materials, not only for having superior mechanical properties, such as high initial strength gain, high fire resistance, and acid and sulfate resistance attacks, but also for be considered eco-friendly materials, considering that, unlike what occurs with Portland cement, the synthesis of geopolymer does not release harmful gases to the environment nor does it depend on an extremely high energy expenditure [2,3,4]. An alternative found to improve environmental issues related to the use of traditional Portland cement is the use of geopolymeric binders with high mechanical performance, such as fiber reinforced ones, which can be processed in different ways, up to the most modern such as 3D printing [5].

However, like other ceramic materials, such as cementitious matrices (pastes, mortars and concrete), geopolymers have low tensile and flexural strength [6]. Furthermore, they are fragile rupture materials, that is, they break abruptly when under the action of a load that reaches the maximum strength of the material [1]. Geopolymer materials also demonstrate a relevant trend of shrinkage, although less than that observed in Portland cement-based materials [7]. Although only a small amount of water, known as “interstitial or structural water”, is needed in the synthesis of the geopolymer gel, another portion of water called “free water” is added to the mixture to make it workable [1]. Loss of free water can cause large deformation due to drying shrinkage [8].

The incorporation of fibers in matrices that present fragility as a mechanical characteristic is an alternative to increase the toughness and ductility of the material, as well as its resistance to bending, since the fibers control the propagation of cracks in the material, under different loading and also in cases where retraction occurs [9]. Studies have already shown that the adhesion between the reinforcement mechanism and the matrix has an influence on the cross-section of the fibers. The result of specimens of cementitious matrices reinforced with fiber before and after rupture, showed a homogeneous distribution of fibers in the matrix, which in this study corroborated with the positive results of mechanical strenght to bending [10].

The use of fibers to reinforce geopolymer composites was first studied by Davidovits, who aimed to produce molds and patterns for the plastics processing industry [7]. Currently, fiber-reinforced geopolymer matrices play an important role as an advanced technological solution in the automobile and aerospace industries and in naval architecture [11]. The most common fibers in geopolymer reinforcement are synthetic fibers such as steel, glass and carbon fibers [12,13,14]. Furthermore, an extensive number of researches have been done in order to analyze the use of polymeric fibers such as polypropylene and polyvinyl alcohol fibers [15,16]. A recent research evaluated different geopolymeric mortars, reinforced with polypropylene fiber (PP), dosed with different silica modules of 0.8, 0.9, and 1.0 and molar ratio of 12, 14, and 16 M, adding PP fibers at 0%, 0.5%, and 1% in relation to the total volume [16]. The results of this research showed an optimal mixture with a molar ratio of 16 M and 0.5% of PP fiber volume, showing satisfactory results with high viscosity, major adhesion, and high mechanical flexural strength. There was also a decrease in voids according to temperature variations, and calcium silicate hydrates (CSH) gels helped to enhance the geopolymerization process [16,17].

The reinforcement of geopolymer composites with natural fibers is slightly recent and is mainly motivated by the growing concern with the maintenance of the environment, as well as the interest in the production of more sustainable building materials. In this scenario, natural fibers are advantageous because they are renewable, biodegradable and non-toxic, in addition to having low density and acceptable mechanical strength [18,19]. Furthermore, the replacement of synthetic fibers by natural fibers is also interesting for economic reasons, given that the production of artificial fibers is an energy-consuming process, unlike the planting and harvesting of natural fibers [11,20,21].

However, it is also necessary to consider that the performance of a geopolymeric material reinforced by fibers, both natural and synthetic, strongly depends on factors such as: the fiber properties, the fiber content used, the geopolymeric precursor, the curing conditions and the age of the sample [1]. Even more important is the bond between the fiber surface and the geopolymer matrix, as a strong bond allows the transfer of charges from the matrix to the fiber, while a weak bond results in a more porous and less resistant material [15,22,23].

Composite materials can present different types of matrices, such as cementitious and geopolymeric, which have a porous appearance in general [24]. The pores existing along the geopolymeric and cementitious matrices, for example, are responsible for the permeability of moisture from the environment into the matrix, also reaching the natural fibers used as reinforcement [25,26]. In addition, the permeability of gases and other external agents that modify the matrix structure, the reinforcement with natural fiber and consequently its durability can occur [27,28]. Studies show that high moisture absorption in composites depends on several factors, such as external and internal temperature, reinforcement orientation, fiber volume fraction, fiber nature (permeable or not), exposed surface area, diffusion, and type of surface protection [29,30,31]. A research evaluated the moisture absorption characteristics of polyester and epoxy matrix composites reinforced with jute fibers, which demonstrated that under the effect of constant humidity and room temperature, the natural fibers contained in this matrix showed an increasing moisture absorption, linked to the increase in fiber volume fraction, while the time required to reach absorption equilibrium decreased with fiber content, which was attributed to the increase in diffusivity in the composite under such conditions [32,33,34].

An important aspect of natural fibers, which are also directed towards their high moisture absorption, is that cellulose-based fibers are called in the literature as polar and hydrophilic, and their compatibility with cementitious and geopolymeric matrices is correlated with interfacial adhesion and absorption [35]. The surface treatment process creates an adhesive on the fiber cell wall, which is responsible for binding the cellulose microfibril together, improving the high moisture absorption aspect seen in the fiber in its natural condition [36]. The hygroscopic behavior is an inherent characteristic of natural fibers that can influence their applications as fabrics and reinforcements in geopolymer composites [37]. The kinetics of water vapor sorption on cotton, filter paper, linen, hemp, jute, sisal fibers were determined in some research using a dynamic vapor sorption device and the results were analyzed using a parallel exponential kinetic model (EKM) [38,39]. The results of the water sorption kinetic curves proved to be slow as well as fast on the fiber cell wall, exhibiting hysteresis between adsorption and desorption. Here, the different natural fibers exhibited a variable dynamics of water vapor sorption behavior, mainly in more porous matrices, such as cementitious and geopolymeric [40].

The use of natural fibers also influences several aspects of composite materials, such as traditional concretes and geopolymers. The most important characteristics for the applications of natural fibers as reinforcement elements are initially related to the type of matrix, such as for example in the case of traditional cementitious matrices, there where is an alkalinity of the medium that interferes with the final properties of the composite, drastically reducing its durability [41]. The type of fiber also influences, in issues such as sugar content and the chemical composition of other elements that can enhance the hydration of the matrix, whether geopolymeric or cementitious, physical and geometric characteristics, fiber addition content, and even the interfacial interaction with the matrix [35,42,43].

Research has already shown that even the way they are arranged and aligned within the cementitious matrix exerts a significant influence on mechanical properties and on the capacity to absorb internal stresses, promoting their reinforcement behavior [44,45]. Another aspect is regarding its release and density at the time of making the mixtures (geopolymers and traditional concrete), which, if performed in the wrong way, can contribute to the increase in the incorporated air content that translates into loss of mechanical properties, this was verified in researches that used coconut fiber in different forms of densification [46]. Another study evaluated the mechanical properties of different composites of natural fibers in polymeric matrices, proving that fiber size is directly related to the mechanical properties of the produced parts [47]. A relevant mechanism for the application of natural fibers is related to the selection method adopted, aiming to categorize and compare the main mechanical and thermal properties of the fibers in general, aiming at their better incorporation, one option is the use of additive manufacturing for these applications [48].

The application of fibers can be made in different materials, for example in the case of short fibers used as reinforcement in composite railway sleepers, so some current research shows potential for the application and development of alternative materials to traditional wooden sleepers, and that to do this, resist mechanical efforts in an adequate way [49]. A study has shown, for example, the importance of using residues in the development of new materials that reduce environmental impacts. This occurs, for example, in the application of residues to make alternative building materials, and natural fibers can be considered agro-industrial wastes [50]. In the case of railway sleepers, glass fibers can be applied, forming a sustainable and durable composite, which must be evaluated for its technological and durability properties, seeing how similar to traditional woods these new materials are effectively [51,52].

Thus, based on the available literature on the subject, this research aims to establish a parallel between the physical and mechanical performance of geopolymer composites reinforced with natural and synthetic fibers, highlighting the main factors that help or hinder the quality of the product final and, thus, contribute to a better understanding of the similarities and differences that exist between both materials. This paper differs from others due to its focus on research that effectively applied natural and synthetics fibers in geopolymeric materials, objectively highlighting issues of mechanical and microstructural properties that interfere with their performance. There is also a relationship defined with cementitious materials in order to compare the results found in geopolymer materials, i.e., there is a future potential for geopolymer materials to advance in terms of use in relation to traditional cementitious materials, so this review article contributes to this panorama.

## 2. Interface between Fiber-Matrix

**Figure 1:**
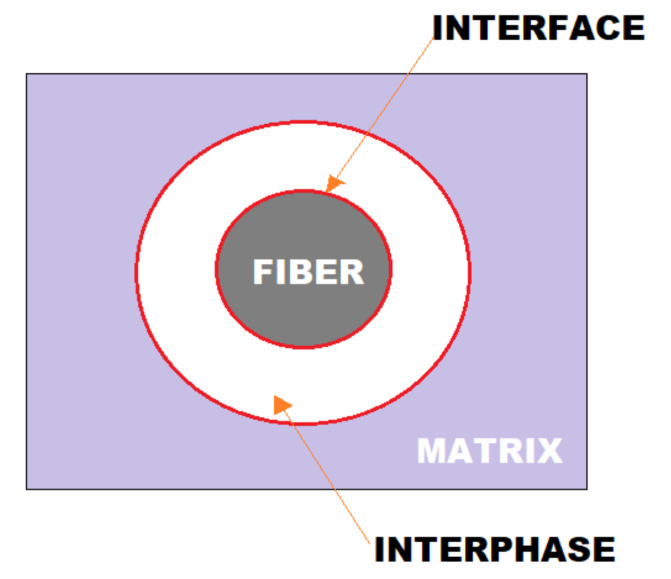
Basic scheme representing interface and interface.

With regard to the physical properties and mechanical behavior of fiber-reinforced geopolymer composites, the fiber–matrix interface, that is, the adhesion between the fiber surface and the geopolymer matrix, plays a crucial role, as it allows the transfer of the applied load from the matrix for the fibers [53]. The intensity of this adhesion, in turn, is determined by the physical and chemical characteristics of the fiber used. Most composite materials are made up of two basic elements, the matrix and a reinforcing material, called filler, always dispersed in the matrix [54]. The matrix, also known as continuous phase, while reinforcement is called dispersed phase. The separation of the two elements takes place through the interface, but even so they have a great adhesion capacity [55]. The matrix has the basic function of protecting the reinforcement from the external environment, preventing the dispersed material (reinforcement) from coming into contact with acidic or corrosive media, as well as keeping the reinforcement in place and transferring tension to it [56]. The composite interface is a hypothetical surface without thickness that joins the reinforcement and the matrix by a certain adhesion mechanism, the interphase can be considered as the region resulting from the interaction between the reinforcement and the matrix with chemical and physical bonds (Figure 1) [57]. One of the main challenges in processing composite materials is to obtain adequate interface and interphase between matrix and reinforcement.

The interphase region can be a diffusion zone, a nucleation zone, a chemical reaction zone, or any combination of these parameters [58]. The interphase region can be formed by depositing a thin layer of polymer on the surface of the reinforcement, for example, or spontaneously developed due to interactions of the matrix with the surface of the reinforcement [58,59]. Both the interface and the interphase have mechanisms that contribute to the mechanical properties of the composite, the main one being the adhesion that can be given by mechanical fitting of the polymer matrix chains in roughness on the reinforcement surface, electrostatic attraction, formation of chemical bonds, or inter-diffusion of materials [59,60].

**Figure 2:**
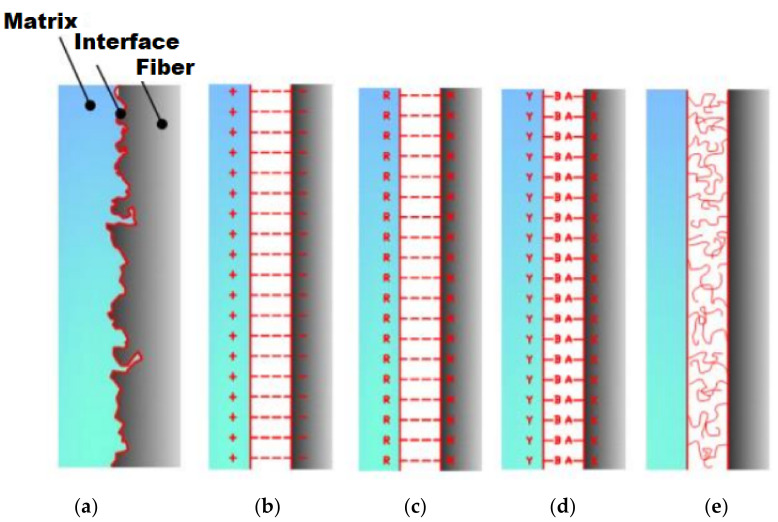
Schematic diagram of adhesion mechanisms at the reinforcement-matrix interface: (**a**) mechanical adhesion; (**b**) electrostatic adhesion; (**c**) chemical adhesion, where R and M represent compatible chemical groups; (**d**) chemical adhesion with application of a coupling agent; (**e**) adhesion by reaction or interdiffusion involving polymers [66].

The adhesion between the matrix and the reinforcement, a mechanism that significantly contributes to the mechanical properties and aids the transfer of loads between the matrix and the reinforcement, can occur by: mechanical engagement of the polymer matrix chains in roughness on the surface of the reinforcement, electrostatic attraction, formation of chemical bonds or interdiffusion of materials [41,61,62]. Achieving a fiber/matrix interface close to the ideal improves the mechanical properties of composites [62]. In mechanical interactions, there is “interlocking” or “keying” between the two surfaces (Figure 2a) where the contraction of the matrix during the curing process is very favorable to this type of bond [63]. They are most effective when the load applied to the material is parallel to the interface, that is, the shear strength must be considered [63]. On the other hand, when the interface is subjected to tensional forces that act perpendicularly, the material strength is very likely to be low, unless there are many angled recesses [64]. Due to these factors, composite materials are considered anisotropic. In many cases, mechanical interaction is not the only phenomenon acting on the material interface, as it can occur in conjunction with other adhesion mechanisms [65].

When electrostatic adhesion between matrix and reinforcement occurs, one surface is positively charged while the other is negatively charged (Figure 2b). This generates an electrostatic attraction between the composite components that will depend on the difference in charges on their surfaces [64]. Electrostatic interactions are short-range and therefore only effective over small distances, on the order of atomic dimensions [65]. Thus, it is essential that there is no contamination on the reinforcement and matrix surfaces, as this factor will determine the effectiveness of this adhesion mechanism [67].

Chemical adhesion is given by chemical bonds, which, in the context of composite science, are bonds formed between chemical groups on the reinforcement surface and compatible groups on the matrix (Figure 2c). The resistance 13 of this type of interaction depends on the type and number of chemical bonds per unit area. Coupling agents are often used for this type of adhesion to occur, such as silanes, which are commonly used by coupling oxide groups on the surface of the glass to bond with molecules of polymeric matrices [68]. At one end (A) of the silane molecule, hydrogen bonds are formed between the oxide groups on the glass and the partially hydrolyzed silane, as the other end (B) reacts with a compatible group on the polymer (Figure 2d) [69]. Finally, adhesion by reaction or interdiffusion occurs when atoms or molecules of the two components of the composite material diffuse at the interface [70]. For interfaces involving polymers, this type of adhesion can, in simple terms, be considered as a tangle of molecules (Figure 2e). In the latter case, the factors that control resistance at the interface are the distance between each entangled molecule, the extent of the entanglement of molecules and the number of molecules per unit area of the interface [70].

Ranjbar et al., analyzed the interfacial bond between geopolymeric pastes and steel microfibers and also with polypropylene fibers. It was possible to identify in the polypropylene fiber a smoother and more regular surface, which does not contribute to a strong adhesion between the fiber and the matrix. On the other hand, steel microfiber, with a much rougher and rougher surface, showed more adherence to the matrix. The performance of the samples in the flexural strength tests proves the relationship between the surface of the fibers and the fiber–matrix interface: slurries reinforced with steel microfibers had higher strengths than the reference for most fiber insertion contents tested, while the samples containing polypropylene fibers showed a decrease in strength for all contents except 4% [1].

In the same article, Ranjbar also analyzed how the affinity that the surface of steel and polypropylene fibers has with water impacts on the fiber–matrix adhesion of the geopolymer paste, since, as geopolymer is a water-based material, the tendency of fiber to absorb (hydrophilicity) or repel (hydrophobia) water can be determinant for a strong bond between the reinforcing fiber and the geopolymer matrix [7]. By investigating the angle of contact with water on the surface of each fiber, it was found that the surface of the polypropylene fiber is hydrophobic, while that of the steel microfiber is hydrophilic. In view of this, and considering the low mechanical performance presented by the pulps reinforced with polypropylene fibers, it is plausible to infer that the regular morphology, combined with the hydrophobic behavior of the fiber surface, led to a weak fiber–matrix interface. Opposite to this, the high mechanical performance of steel microfiber reinforced pastes is equally related to the rugged morphology and the hydrophilic behavior of the fiber surface.

On the other hand, with regard to geopolymer composites reinforced by natural fibers, although the affinity with water and the morphology of the fiber surface are still important factors, other characteristics can be decisive for the fiber–matrix adhesion [71].

Natural fibers, in terms of chemical composition, are biopolymer composites formed mainly by cellulose (40–60%), semi-cellulose (20–40%), and lignin (10–25%) [72]. Table 1 shows the contents of these three components in different natural fibers, while the other components such as impurities, ash, and extractives are found in small percentages and are not significant for discussion about natural fibers in this type of matrix, being the basis of difference for the 100% total. One of the main limitations to the use of natural fibers as a reinforcement in geopolymer matrices is directly related to the chemical composition of the fibers, since non-cellulosic organic components, such as semi cellulose and lignin, are sensitive to highly alkaline environments [18] and they tend to degrade under these conditions, which not only decreases the fiber’s compatibility with the geopolymer matrix, but also causes a reduction in the composite’s properties over time [18,73].

Ye et al. [72] studied the effects of cellulose, semi cellulose, and lignin in geopolymer mortars produced with metakaolin. The research observed that the addition of the semi cellulose content in the sample delayed the hardening of the mortar due to the degradation of this substance in an alkaline environment, which generates carboxylic acid as a product, which, in turn, inhibits geopolymerization reactions. The results presented in the density tests also indicated that the addition of lignin and, mainly, of semi-cellulose in the samples caused an increase in porosity, a behavior that was accentuated with the increase in the content of incorporated lignin and semi-cellulose. On the other hand, the study found that cellulose does not undergo significant degradation in an alkaline medium and also allows a good adhesion between the fiber and the geopolymer matrix, as a result, the samples containing cellulose presented the densest structures during the tests [72].

Furthermore, with regard to the use of natural fibers as reinforcement in geopolymer matrices, fiber–matrix adhesion can, as occurs with synthetic fibers, be affected by the hydrophilic and hydrophobic behavior of the fibers [74]. The presence of hydroxyls and other polar groups causes natural fibers to become hydrophilic by nature, however, it is necessary to consider that cellulosic fibers have high moisture absorption and little dimensional stability, as they swell in contact with water. This behavior leads not only to the weakening of the fiber–matrix adhesion, but also to the formation of cracks, resulting from the drying of the composite, and a reduction in mechanical performance [74,75].

On the other hand, the presence of inorganic compounds, such as lignin, on the surface of natural fibers also reduces the adhesion between the fiber and the hydrophilic geopolymer matrix, which in turn negatively affects the mechanical performance of the composite [18].

Considering all the factors exposed here, to be overcome in order to obtain a geopolymer composite with satisfactory fiber–matrix adhesion, in several studies, natural fibers are subjected to chemical pre-treatments aimed at promoting changes in the fiber surface and composition fiber chemistry so that the fiber–matrix interface is optimized.

## 3. Surface Treatment of Natural Fibers

Fiber pre-treatment consists of a set of processes whose purpose is to improve the adhesion between the surface of the fibers and the geopolymer matrix and, in this way, ensure that the transfer of tension from the matrix to the fiber occurs properly. The pre-treatment exposes reactive groups on the fiber surface, facilitating bonding with the matrix. Furthermore, it not only removes impurities, making the surface cleaner and more rugged, but also reduces the fiber’s water absorption capacity [76,77].

There are a large number of fiber surface treatment processes described in the literature, among which the most common is mercerization, or alkaline treatment, which consists of immersing the fibers in an alkaline solution, usually based on hydroxide sodium (NaOH) [78,79]. The concentration of the solution, the duration of the treatment and the temperature at which the process takes place are determining factors for the efficiency of this procedure and are guidelines in several studies [80,81].

**Figure 3:**
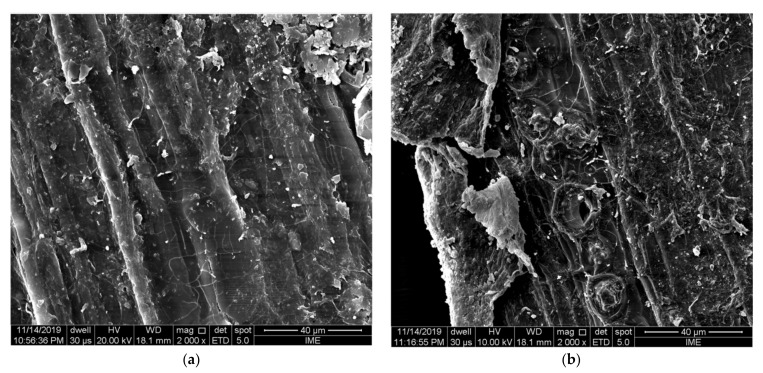
Fiber defibrillation: (**a**) untreated; (**b**) with treatment with NaOH solution [43].

In this type of treatment, the alkaline solution is responsible for breaking hydrogen bonds that support the entire structural system of the fiber, promoting an increase in roughness [77,82]. In addition, it also removes semi cellulose, lignin, and other non-cellulosic substances from the fiber, such as waxes and oils [18,77,82,83]. These substances, in the fiber, work as binders, and their removal, through alkaline treatment helps to separate fiber agglomerates into elementary fibers [18,84]. The defibrillation of fiber clusters, as well as the reduction in fiber diameter, consequences of mercerization, directly implies an increase in the specific contact area between the fiber and the geopolymer matrix. Thus, there is an increase in barriers against the expansion of cracks in the matrix. This represents a gain in the flexural strength of composites [18]. This process is shown in Figure 3.

Lazorenko et al. [64] studied the effects of incorporating untreated flax fibers into fly ash-based geopolymer matrices. For this, samples were produced containing 0.25%, 0.50%, 0.75%, and 1% of waste. Through mechanical tests, it was clear that the optimum fiber introduction content corresponds to 1%. There was an increase in flexural strength (4.0MPa in the reference sample and 4.9 MPa in the sample containing 1% fiber) and there were also significant improvements in the post-breaking behavior of the samples, which started to show more ductility with the addition of fibers. However, the compressive strength test showed the negative effect that the insertion of the fiber produced in the composite, given the drop in strength from 44MPa in the reference sample to 32.5MPa in the sample with 1% fiber. The authors associated this behavior with the possible low fiber–matrix adhesion of the composite, or even the low fiber resistance to loading [64,85].

In a complementary article to the aforementioned, Lazorenko et al. [64] analyzed the impacts of applying different pre-treatments on flax fibers for the production of fly ash-based geopolymer composites. For comparison purposes, the fiber incorporation contents of the preceding article were maintained, i.e., 0.25%, 0.50%, 0.75%, and 1%. The fibers were subjected to three types of pre-treatments separately: mechanical cleaning of impurities, alkaline treatment in aqueous solution with 5% NaOH for 60 min and alkaline treatment, also in aqueous solution with 5% NaOH for 30 min, combined with ultrasonic treatment for another 30 min. The characterization by infrared spectroscopy revealed that the alkaline treatment, as well as the alkaline/ultrasonic treatment was more effective in removing semi cellulose and other non-cellulosic substances that impair the fiber–matrix interface interaction. The flexural strength tests showed, once again, the superior efficacy of the alkaline treatment and the alkaline/ultrasonic treatment. The latter in particular obtained the highest strength values, obtaining 5.4 MPa of flexural strength for samples with 1% fibers, an increase of 35% compared to the unreinforced geopolymer (4.0 MPa). The samples containing 1% of fibers treated through mercerization had a resistance of 4.6 MPa, which represents a 15% increase in relation to the reference [86].

The research also found the transition from fragile rupture, in non-reinforced samples, to a much more plastic rupture in samples with 1% of fibers submitted to alkaline-ultrasonic treatment. Therefore, the importance of pre-treatments in improving the adhesion between the fiber surface and the geopolymer matrix, and consequently in increasing the mechanical strength and improving the post-rupture behavior of the composite, became clear [87].

Chemical modification of cellulose fiber surfaces can lead to increased viscosity, thus reducing the shear dilution effect expected during processing [88]. Therefore, the fiber type, its modification and functionality can greatly improve the fiber-matrix interfacial interaction, leading to improved performance properties, especially in geopolymer matrices [89].

The processing step of these materials is fundamental, as it is related to their final performance [90]. For the surface modification of fibers, there are several treatments that can be carried out in addition to immersion in NaOH, such as alkaline treatment and calcium hydroxide solution, potassium hydroxide (KOH) or even heat treatment. Some researchers have evaluated the effect of treatments in other alkaline solutions, with a view to improving performance. However, some more recent researches also suggest the possibility of changing the matrix condition, which can be performed with the stabilization of materials inserted in the geopolymeric mixture [12].

Alkaline treatment by calcium hydroxide Ca(OH)_2_ is efficient in the total or partial removal of hemicellulose and lignin. It leads to the formation of a layer of CaCO_3_ deposited on the fiber, preventing defibrillation. This keeps the crystal structure of the fiber intact, keeping the cellulose [91].

Santos et al. [92] demonstrated that, comparing the two treatments, NaOH and Ca(OH)_2_, the treatment with calcium hydroxide proved to be less aggressive to the surface of a piassava fiber than the treatment with NaOH. They also found that the greater weight gain of the fiber handled with NaOH indicates that a greater amount of water penetrated into its structure, consequently defibrillating the fiber and reducing its size, as the fiber handled with Ca(OH)_2_ did not defibrillate.

Few differences were observed in the literature when comparing the mentioned alkaline treatments with KOH [93,94]. Moghaddam and Mortazavi [95] analyzed that alkaline treatment with potassium hydroxide makes the fibers achieve higher characteristics of linear density and tensile properties compared to sodium hydroxide.

Finally, in the heat treatment, the fibers are cleaned and dried and then heated, reaching a temperature of up to 200 °C. The heat treatment promotes a reduction in the hemicellulose and lignin contents of the fibers, increasing their stiffness [96].

Comparing the application of natural and synthetic fibers in geopolymeric concretes, one of the main challenges is the need for pre-treatment of natural fibers for their application in high alkalinity matrices, as is the case of geopolymeric ones [12]. This treatment process generates increased costs and makes it difficult to standardize the process, however the high abundance of natural fibers that currently exist makes this material widely available and economical, and often with superior strength characteristics than synthetic fibers [97]. On the other hand, the use of synthetic fibers brings greater reliability to the composite, due to the regularity of its production process, in addition to dispensing with surface treatments and/or changes in the geopolymer matrix [98]. A comparative study of the application of natural and synthetic fiber in a geopolymer concrete showed that, in technological terms, the natural fiber had better capacity to absorb tension in the matrix, while the synthetic fibers were more economical in the process due to no need for prior treatment [99,100]. In the fresh state properties, the synthetic fibers showed adhesion advantages, due to the greater regularity of the surface and the non-absorption of water impregnated in the matrix, a behavior that natural fibers have difficulty, even after the treatment process [99,100]. Therefore, it is concluded that the use of natural fibers as reinforcement in structures with high structural reliability still needs to be studied further [101].

Regarding fresh state properties, such as workability, the addition of steel fiber to geopolymer concrete increases tensile strength, toughness, bending, fracture, shrinkage, and crack reduction. However, there are disadvantages, such as the potential increase in voids and compatibility of materials [12]. [102] indicate values around 0.75% of the steel fiber volumetric fraction to be inserted in the geopolymer matrix, with 0.65% being a minimum value for good workability.

## 4. Mechanical Performance of Fiber Reinforced Geopolymer Composites

Regarding the workability of fiber-reinforced geopolymer matrices, studies available in the literature report a similar behavior between natural and synthetic fibers: both types of fiber cause a decrease in the fluidity of the geopolymer mixture, a trend that increases with the increase in the content of incorporation of fiber into the composite and it does not depend on which fiber is being used [7,73,100,103,104]. This behavior is caused by a large shear force between the fibers and the geopolymer matrix, which prevents fluidity and makes the mixture more consistent, therefore, less workable. Increasing fiber content leads to increased strength, which further reduces workability [7].

Therefore, the insertion of fibers in geopolymer composites can pose a risk to the density of the material, as the poor distribution of fibers in the matrix, in its fresh state, promotes the appearance of fiber agglomerates and voids trapped between them [7]. In the fresh state, these voids are filled with water, in the hardened state, in turn, with the evaporation of water, the composite becomes porous and, consequently, less dense. This behavior is also accentuated with the increase of the incorporated fiber content and, in addition, it directly influences the mechanical strength of the material [105].

**Figure 4:**
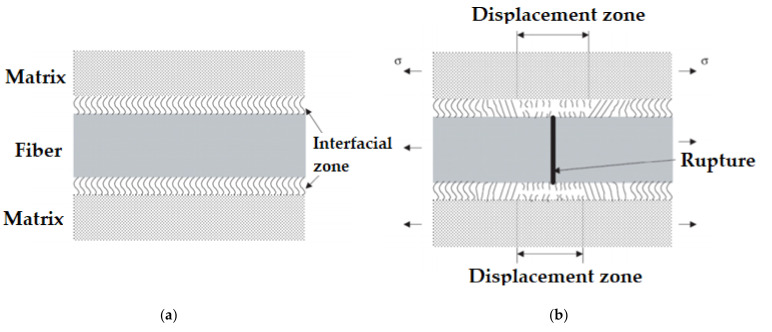
Schematic of the microstructure of the fiber–matrix interface: (**a**) before load application and without fiber breakage; (**b**) after application of the load, with fiber breakage.

Among the mechanical properties that influence geopolymeric matrices, the resistance that occurs in the interface region is directly related to the atomic, molecular or even polar bonds that occur between the matrix and the reinforcement fiber [106]. This phenomenon can be exemplified according to the scheme in Figure 4, which shows the fragmentation process on a molecular scale, as the application of a charge begins, putting tension on the molecular connection lines, which end up tensioning [8]. When the load is continued, the fiber breaks, and then some connections in the fracture area are broken almost simultaneously. At this stage, the fiber breaks into two fragments, in addition to a void in the rupture region (break gap) [107]. With these connections breaking close to the fracture region, the connections closest to these end up being maintained, even if they remain tensioned, while the connection lines further away from the fiber rupture are not much affected by the applied effort, maintaining the original condition of regularity and balance [108].

In Figure 4a,b, it is also possible to observe the interface of the material in the condition before and after applying the load, defining the displacement zone as a region where the molecular bonds end up breaking, including the “break gap” of the fiber, which is symmetric in both sides of the fiber.

Alomayri et al. [103] investigated the physical characteristics of geopolymer pastes reinforced with cotton fibers (insertion contents: 0.3%, 0.5%, 0.7%, and 1%), reported that samples containing 0.7% and 1% of fibers showed significant loss of workability, which made molding the specimens difficult. To solve this problem, an amount of free water was added to the mixture to allow for molding. The tests later showed that these samples had high levels of porosity, and consequently low density. It was possible to conclude that the high content of fiber insertion combined with the necessary addition of free water to the mixture resulted in fiber agglomeration and water confinement among them. Evaporation of water led to high levels of porosity. Flexural strength tests confirmed the low mechanical performance of these samples [103].

Furthermore, the physical characteristics of the insulated fibers also interfere with the final density of the composite, that is, a fiber that has a low-density value by itself also contributes to the reduction of the final material density [73].

The reduction of shrinkage suffered by pure geopolymers is one of the pillars that support the practice of using fibers in the reinforcement of geopolymer composites. The mechanism that promotes this behavior, in turn, is the same for synthetic and natural fibers [7]. Firstly, part of the retraction energy is canceled as a result of tensions existing in the matrix, which are caused by the pressure and friction existing in the fiber–matrix interface. Furthermore, when faced with a fissure in the geopolymer matrix, the fibers act as bridges, which cross the fissures and retard their growth [7].

The efficiency of this process is, therefore, linked to the intensity of the interaction between the fiber and the geopolymer matrix. Ranjbar et al. [7] analyzed the influence of polypropylene fibers on the physical and mechanical properties of geopolymer composites produced from fly ash. From the results obtained in the tests, combined with the visual examination of the samples, the research found that low fiber contents in the composite (between 0.5% and 3%) reduced the shrinkage suffered, while greater fiber insertions (4% and 5%) increased the retraction values considerably. Even so, it was possible to visualize cracks in the samples with contents between 0.5% and 3%, and this is due to the weak adhesion that the polypropylene fiber has with the geopolymer matrix, which does not allow the shrinkage stresses to be overcome [7].

**Figure 5:**
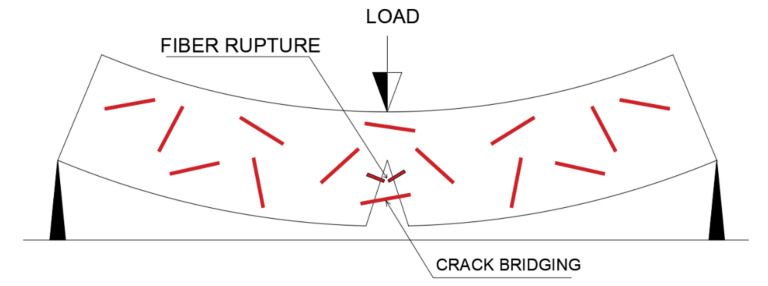
Fiber organization scheme in a crack.

The ability of the fibers to serve as bridges between the cracks, slowing their growth is also fundamental for the evolution of the composite’s flexural strength. When a crack hits the fiber, a certain amount of energy is needed to disjoin the fiber from the matrix, overcoming adhesion, and propagate through the composite [109,110]. In this process, the fiber can be broken or pulled from the geopolymer matrix, a process illustrated in Figure 5. According to the results obtained in the research by Noushini et al. [104], the situation that represents the greatest gain in bending strength occurs when the fiber is pulled from the matrix, as a result of the additional energy that is needed to overcome the friction between the fiber and the matrix, which is not dissipated in case of fiber rupture [104,110].

The application of natural fibers in specimens lead to a lower elastic modulus than synthetic fibers, which ends up leading to lower mechanical resistance to compression. Compression mechanical rupture has a direct influence on fiber geometry, density, microfibril angle and moisture absorption capacity [12]. On the other hand, a research that investigated the use of sisal and coconut fiber in geopolymers with a high content of fly ash concluded that the addition of 0.5% resulted in this case in improved mechanical compression strength [100]. What has been observed in various studies is that the addition of natural fibers, depending on their orientation, can improve the capacity to absorb and transmit internal efforts, which makes the specimen more ductile [12]. For flexural strength, a property applied to geopolymer mortars with prismatic specimens, the length of the fibers and their adhesion and anchorage to the geopolymer matrix, allows an increase in the final values of the flexural strength [100]. In general, the application of natural fibers as reinforcement of these composites provides the reduction of cracks and their propagation, due to the absorption of tensions by the fibrous reinforcement element [111].

Wongsa et al. [100] established a parallel between the physical and mechanical performance of geopolymer mortars with fly ash containing high calcium content that used coconut and sisal fibers as reinforcement, compared to mortars reinforced with glass fibers. The fiber insertion rates adopted by the research were 0.5%, 0.75%, and 1%, which defined the mortar workability limit. Above this limit, the mixture became very consistent and difficult to mold. The research found that the flexural strength of composites grew proportionally to the increase in the presence of fibers, with the best results belonging to samples reinforced with sisal and coconut fibers, confirming the efficiency of the researched natural fibers and their potential to replace synthetic fibers (in question, the fiberglass) in the reinforcement of geopolymer matrices without strength deficit for the composite [100].

Alomayri et al. [103], analyzing the performance of geopolymeric pulps reinforced with cotton fibers to bending effort, concluded that, among the insertion contents evaluated in the research (0, 0.3%, 0.5%, 0.7%, and 1%), pulps with 0.5% of cotton fibers showed the best results, obtaining 11.7 MPa of flexural strength (28 days) compared to 10.4 MPa achieved by the reference paste. Larger insertions resulted in a reduction in strength and, according to the authors, this behavior is linked to poor dispersion of the fibers in the composite, resulting in fiber agglomeration that impairs the adhesion between the fibers and the matrix and acts as a stress concentration, reducing flexural strength [103].

Haddaji et al. [73] examined the physical and mechanical behavior of geopolymer pastes produced from the mixture of metakaolin and waste from phosphate mining, with the incorporation of polypropylene and glass fibers. It was found that the insertion of fibers improves the performance of composites to bending stress, especially in samples reinforced with polypropylene fibers, where the increase in strength was more expressive, reaching 9.62 MPa of strength at 28 days, with 1% of fibers. The same content of glass fiber incorporation gave the composite a strength of 5.49 MPa, at 28 days [73].

The crack propagation control mechanisms that give fiber-reinforced geopolymer matrices better resistance to bending stress, as well as reducing shrinkage levels, are also responsible for increasing the ductility and toughness of the composite [100]. In fact, several studies claim that fiber-reinforced geopolymer composites, after breaking due to loading, manage to maintain their shape, which characterizes a plastic break. On the contrary, in the absence of fiber reinforcement, the composite breaks entirely, presenting a fragile break [100].

Toughness represents the measure of energy absorbed by a composite during plastic deformation. Materials with low tenacity present fragile and abrupt rupture, on the other hand, higher levels of tenacity guarantee prolonged ruptures that show signs of occurrence, alerting the failure of the composite, which makes the material safer from the perspective of civil construction [104]. By restricting the width of the cracks in the composite, and limiting their propagation, the fibers improve the material’s energy absorption capacity (tenacity), and consequently, make it more ductile [7].

**Figure 6:**
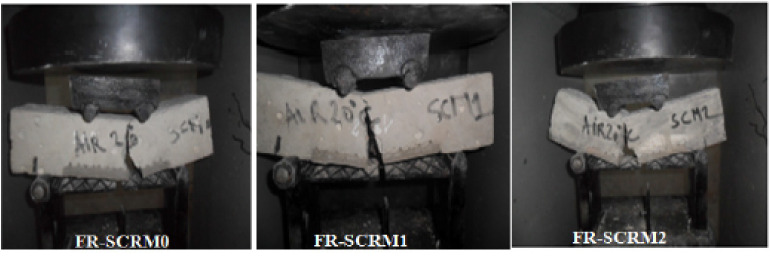
Breakage of specimens with fiber [112].

Once a crack is formed in the composite, a drop in strength occurs, along which bending stresses are transferred from the matrix to the fibers. Figure 6 shows the differences between the failures of composites with fibers. The “bridge effect”, which can be seen in figures, prevents the composite from breaking abruptly and transfers the stress to other areas. Once stresses in other areas have again exceeded the material’s capacity, new cracks form and the process repeats. There is, however, an active crack, which grows and causes the sample to fail, despite the existence of several other secondary cracks [2].

In another research, there was a study of the mechanical behavior of geopolymer composites with high calcium content reinforced with sisal, coconut and glass fibers. Wongsa et al. [100] also found an increase in the ductility of the reinforced samples compared to the reference sample, this time, however, with the specimens subjected to compression stress.

Although the purpose of using fibers in the synthesis of geopolymer composites is to promote an increase in ductility and control the propagation of cracks, and not an increase in compressive strength [74], studies indicate that fibers do not necessarily lead to a decrease in compressive strength, and often improve the performance of composites when compressed [113,114].

Alomayri and Low, [115] when investigating the mechanical performance of geopolymer pastes reinforced with alkaline treated cotton fibers, found that the addition of 0.5% of fibers in the composite allowed the compressive strength to reach 46 MPa at 28 days, compared to 19.1 MPa of the reference folder [115].

In turn, Korniejenko et al. [11] produced geopolymer composites based on fly ash and reinforced with the introduction of 1% of the fibers of sisal, cotton, corn and raffia. The results observed in the compressive strength tests showed improved strength for all fibers tested, except for the raffia fiber, which reached only 13.66 MPa of strength at 28 days and was below the reference (24.78 MPa). The best performance was achieved by samples reinforced with corn fibers, which reached a strength of 31.36 MPa at 28 days [11].

Bhutta et al. [116] investigated the performance of fly ash-based geopolymer mortars, with the reinforcement of different types of fibers, including: steel, PVA and polypropylene, at insertion contents of 0.5% and 1% [116]. Furthermore, with the purpose of evaluating the influence of thermal cure on the strength of the composites, the samples were subjected to periods of 4 or 24 h at a temperature of 80 °C. At 28 days, the maximum strengths reached by the composites reinforced with steel fiber, PVA, and polypropylene in the compressive strength test were 50.3, 42.98, and 43.15, respectively. The research found that the compressive strength of the composites ranged from 22 to 28 MPa for samples subjected to ambient curing, and between 35 and 50 MPa for thermally cured samples. Furthermore, the variation in the volume of fibers inserted in the composites (0.5 or 1%) did not show a significant influence on the compressive strength [116].

Using the same fibers described above (steel, PVA and polypropylene), in the contents of 0.4%, 0.8%, and 1.2%, in geopolymer composites also submitted to thermal cure at 80 °C for 24 h, Al-mashhadani et al. [117] obtained, at 28 days, compressive strengths of 62.52, 63.06 and 60.97 MPa. Samples reinforced with steel fibers and PVA reached their maximum strengths with 1.2% fiber insertion. The samples with polypropylene fibers, on the other hand, showed a decreasing trend of strength in relation to the fiber content, obtaining the maximum strength for the minimum volume of fibers present in the composite [117].

There are, in the literature, conflicting results regarding the compressive strength of fiber-reinforced geopolymer composites. It is important to recognize, however, that this mechanical property is also influenced by other parameters, such as: composite design, type of fiber used, fiber content inserted in the composite, curing conditions, and possibly even the size of the samples [116].

**Figure 7:**
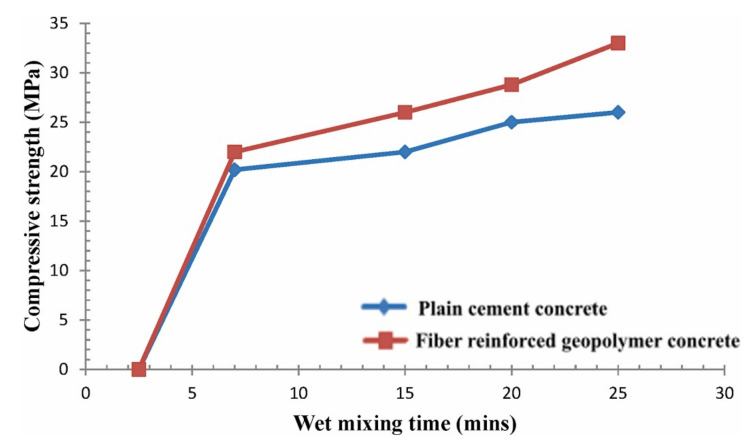
Variation of mixing time on the mechanical compressive strength of traditional and geopolymer concrete [118].

The incorporation of fibers in geopolymeric matrices has, in general, the function of increasing the mechanical strength to bending and energy absorption, and can be incorporated in the form of threads, filaments and nanoparticles. The incorporation, for example, of shorter fibers randomly in the cementitious matrix, tends to increase the tenacity and ductility, in addition to the potential reduction of cracks [41]. Some researches evaluate the curing conditions of specimens and their influence on mechanical parameters. One of these studies evaluated the mechanical resistance to compression as a function of a cure at a temperature of 90 °C at different times of wet mixing, compared to traditional concrete (in blue) with the fiberized geopolymer concrete (in red), as seen in Figure 7.

There is also a comparison made between the amount of fibers to be added in a geopolymer matrix and its influence on the properties of the fresh and hardened state. As for the fresh state, it is known that the excessive addition of fibers causes a blockage of the internal flow of the matrix, increasing the porosity, which consequently reduces its mechanical strength. Thus, the determination of the ideal amount of fibers influences workability and compactness, affecting interfacial adhesion. As for mechanical stiffness, there is a series of solutions in the literature related to fiber treatments, which seek to improve their adhesion and stress absorption. The incorporation of steel fiber in a geopolymer matrix was carried out in a research that found an increase of 54% in the mechanical strength to compression, for the addition of 1%. These researchers also evaluated a hybrid reinforcement, with steel fiber and polypropylene, both in 1%, which promoted a 105% increase in mechanical strength. This large increase in mechanical strength to compression was attributed to hydrophilic issues of this type of fiber and to smaller surface defects, which reduced the debonding effect between the fiber and the matrix [119].

## 5. Discussion Section

In order to compare some results obtained in the international literature, six studies were selected to evaluate some technical parameters of geopolymers, three of which were reinforced with natural fibers (such as jute, sisal, and cotton) and three with synthetic fibers (such as glass, steel, and carbon). The results are shown in Table 2.

The results presented in Table 2 indicate that the application of natural fibers, in all conditions, took place with a cure of 28 days after molding the geopolymeric specimens and with the fibers treated with a NaOH solution. Thus, it can be observed that the fibers of sisal and jute nominal columns with similar mechanical strength to compaction and bending, this corroborates some studies that highlight the proximity of the configurations of both fibers, regarding defined properties and applications [42]. Meanwhile, a cotton fiber has higher values of mechanical strength. This is due to the fact that the dispersion and size of this fiber provided greater adherence to the geopolymer matrix, and also because they are less affected by the existing alkaline environment. A problem observed in the exploratory studies consulted refers to the high dispersion of the results found, especially when related to the use of natural fibers. This high dispersion is justified by the heterogeneity of the raw material and natural processes that are related to its use [123].

In the case of synthetic fibers, these presented values much higher than natural fibers, mainly related to the high efficiency that these fibers play in the interfacial region and in the adhesion between the matrix and the fiber itself. The result of the flexural strength of the Alumina-coated steel was very high and close to compression, which can be explained according to the literature by the compactness effect that occurred in the matrix and the high efficiency in the added percentages [121]. A positive point of synthetic fibers is due to their regularity of constitution due to the mechanized production process, this was verified in the smaller dispersion of the absolute values observed in the studies. Carbon fiber represents a high potential in terms of application advantages and geopolymer systems, mainly for structural purposes [122,124].

Comparatively, the application of synthetic fibers presents advantages of technological results, however the production costs and the environmental impacts of these processes are still high. Meanwhile, the use of natural fibers, as long as they are treated, has high potential, suitability to technical standards, as well as low cost, due to the abundance of these fibers practically all over the world. In many countries, natural fibers are treated as waste, so their application in new composite materials is interesting [125].

## 6. Conclusions

The use of fibers in the production of geopolymer composites is important so that the material’s mechanical deficiencies, especially its low flexural strength and brittleness, can be overcome. In addition, the current concern with the environment arouses interest in the production of more ecological materials. In this context, the use of natural fibers in the reinforcement of geopolymeric matrices presents itself as an important alternative, as it represents a solution for the two problems mentioned above.

However, it is necessary to consider the physical and mechanical behavior of composites reinforced with natural fibers to judge the feasibility of using this material. In order to elucidate this issue, this article sought to establish a comparison between the physical and mechanical performance of geopolymer matrices reinforced with natural fibers compared to those using synthetic fibers, having as reference several studies available in the literature.

Throughout the article, the importance of adhesion between the fiber surface and the geopolymer matrix for the proper transfer of stresses from the matrix to the fibers became clear. This characteristic is directly related to fiber characteristics, and therefore varies easily. Natural fibers, however, are sensitive to the alkaline environment and have substances on their surface that do not favor good adhesion with the matrix. Fiber pretreatment is necessary to solve this problem and optimize composite performance.

Regarding the mechanical performance, a small deficit of resistance of composites reinforced with natural fibers in relation to those with synthetic fibers was observed. It must be considered, however, that most of the articles that served as the basis for its development did not use the pre-treatment of natural fibers in their research, as already explained, essential for composites reinforced with natural fibers to reach their maximum performance. Better analysis can still be done in this regard.

Given the above, it is understood that natural fibers, like synthetic ones, overcome the problems that underlie the use of fibers in geopolymer matrices: low bending strength, high rate of crack propagation and fragility. Despite the existence of a small gap between the strengths achieved by the two composites, the pre-treatment of natural fibers combined with other practices, such as the optimization of the geopolymer trace and curing conditions, can equal the performances. Therefore, natural fibers can replace synthetic ones without damaging the material’s performance.

Finally, we can conclude with the results of this research that the application of fibers in geopolymer materials brings great technological advantages and that, in addition, in the case of natural fibers there is a huge availability of this material and a low extraction cost, which will certainly help to disseminate and popularize geopolymer materials for applications that require greater efforts, especially in respect of mechanical flexural strength. The literature discussed in this document contributes to the knowledge of fiber processing and treatment techniques for application in geopolymers, in order to make this material more competitive with traditional concrete.

## Figures and Tables

**Table 1:** Cellulose, semi cellulose and lignin contents in natural fibers.

Natural Fiber	Cellulose (%)	Semi Cellulose (%)	Lignin (%)	Total (%)
Cotton	89.7	1.0	2.7	93.4
Linen	80.0	13.0	2.0	95.0
Hemp	74.1	7.6	2.2	83.9
Sugar cane	51.8	27.6	10.7	90.1
Bamboo	54.6	11.4	21.7	87.7
Coconut	51.3	11.7	30.7	93.7
Wheat Straw	38.0	36.0	22.0	96.0

**Table 2:** Comparative results of some researches with geopolymers reinforced with natural and synthetic fibers.

Parameters	Natural Fibers						Synthetic Fibers					
	Jute [42]		Sisal [42]		Cotton [120]		Alumina-coated Steel [121]		Polypropylene and Steel fibers [1]		Carbon Fiber [122]	
	wt.% Fibers	Result	wt.% Fibers	Result	wt.% Fibers	Result	wt.% Fibers	Result	wt.% Fibers	Result	wt.% Fibers	Result
Compressive strength (MPa)	0	12.80	0	12.80	0	19.0	0	67.50	0	30.00	0	23.40
	0.50	16.10	0.50	16.70	0.30	27.2	0.50	69.00	0.50	40.50	0.50	26.50
	1.00	17.20	1.00	17.50	0.50	46.5	0.75	72.00	1.00	42.10	-	-
	1.50	21.20	1.50	18.40	0.70	35.0	1.00	69.50	2.00	51.60	-	-
	2.00	18.50	2.00	18.60	1.00	28.1	1.50	69.00	3.00	54.70	-	-
Flexural strength (MPa)	0	0.85	0	0.85	0	0.72	0	66.50	0	3.50	0	4.40
	0.50	1.54	0.50	1.30	0.30	1.20	0.50	67.50	0.50	7.80	0.50	5.50
	1.00	2.10	1.00	1.74	0.50	1.30	0.75	69.50	1.00	11.00	-	-
	1.50	2.50	1.50	2.40	0.70	1.50	1.00	70.00	2.00	18.40	-	-
	2.00	3.30	2.00	2.58	1.00	2.10	1.50	71.50	3.00	23.45	-	-

## Data Availability

Not applicable.
